# Systematic Review: Genetic, Neuroimaging, and Fluids Biomarkers for Frontotemporal Dementia Across Latin America Countries

**DOI:** 10.3389/fneur.2021.663407

**Published:** 2021-06-24

**Authors:** Claudia Duran-Aniotz, Paulina Orellana, Tomas Leon Rodriguez, Fernando Henriquez, Victoria Cabello, María F. Aguirre-Pinto, Tamara Escobedo, Leonel T. Takada, Stefanie D. Pina-Escudero, Oscar Lopez, Jennifer S. Yokoyama, Agustin Ibanez, Mario A. Parra, Andrea Slachevsky

**Affiliations:** ^1^Latin American Institute for Brain Health (BrainLat), Universidad Adolfo Ibanez, Santiago, Chile; ^2^Center for Social and Cognitive Neuroscience (CSCN), School of Psychology, Universidad Adolfo Ibanez, Santiago, Chile; ^3^Trinity College, Global Brain Health Institute, Dublin, Ireland; ^4^Memory and Neuropsychiatric Clinic (CMYN) Neurology Department, Hospital del Salvador and Faculty of Medicine, University of Chile, Santiago, Chile; ^5^Neuropsychology and Clinical Neuroscience Laboratory (LANNEC), Physiopathology Department - Institute of Biomedical Sciences (ICBM), Neuroscience and East Neuroscience Departments, Faculty of Medicine, University of Chile, Santiago, Chile; ^6^Geroscience Center for Brain Health and Metabolism (GERO), Santiago, Chile; ^7^Cognitive and Behavioral Neurology Unit - Department of Neurology, University of São Paulo, São Paulo, Brazil; ^8^Global Brain Health Institute (GBHI), University of California San Francisco (UCSF), San Francisco, CA, United States; ^9^UCSF Department of Neurology, Memory and Aging Center, UCSF, San Francisco, CA, United States; ^10^Department of Neurology, School of Medicine, University of Pittsburgh, Pittsburgh, PA, United States; ^11^Department of Psychiatry, School of Medicine, University of Pittsburgh, Pittsburgh, PA, United States; ^12^Cognitive Neuroscience Center (CNC), Universidad de San Andrés, & National Scientific and Technical Research Council (CONICET), Buenos Aires, Argentina; ^13^School of Psychological Sciences and Health, University of Strathclyde, Glasgow, United Kingdom; ^14^Department of Neurology and Psychiatry, Clínica Alemana-Universidad del Desarrollo, Santiago, Chile

**Keywords:** frontotemporal dementia, genetics, neuroimaging, fluid biomarkers, Latin America

## Abstract

Frontotemporal dementia (FTD) includes a group of clinically, genetically, and pathologically heterogeneous neurodegenerative disorders, affecting the fronto-insular-temporal regions of the brain. Clinically, FTD is characterized by progressive deficits in behavior, executive function, and language and its diagnosis relies mainly on the clinical expertise of the physician/consensus group and the use of neuropsychological tests and/or structural/functional neuroimaging, depending on local availability. The modest correlation between clinical findings and FTD neuropathology makes the diagnosis difficult using clinical criteria and often leads to underdiagnosis or misdiagnosis, primarily due to lack of recognition or awareness of FTD as a disease and symptom overlap with psychiatric disorders. Despite advances in understanding the underlying neuropathology of FTD, accurate and sensitive diagnosis for this disease is still lacking. One of the major challenges is to improve diagnosis in FTD patients as early as possible. In this context, biomarkers have emerged as useful methods to provide and/or complement clinical diagnosis for this complex syndrome, although more evidence is needed to incorporate most of them into clinical practice. However, most biomarker studies have been performed using North American or European populations, with little representation of the Latin American and the Caribbean (LAC) region. In the LAC region, there are additional challenges, particularly the lack of awareness and knowledge about FTD, even in specialists. Also, LAC genetic heritage and cultures are complex, and both likely influence clinical presentations and may modify baseline biomarker levels. Even more, due to diagnostic delay, the clinical presentation might be further complicated by both neurological and psychiatric comorbidity, such as vascular brain damage, substance abuse, mood disorders, among others. This systematic review provides a brief update and an overview of the current knowledge on genetic, neuroimaging, and fluid biomarkers for FTD in LAC countries. Our review highlights the need for extensive research on biomarkers in FTD in LAC to contribute to a more comprehensive understanding of the disease and its associated biomarkers. Dementia research is certainly reduced in the LAC region, highlighting an urgent need for harmonized, innovative, and cross-regional studies with a global perspective across multiple areas of dementia knowledge.

## Introduction

Dementia in Latin America and the Caribbean (LAC) countries has become a major challenge (1–3). The World Neurology Congress has highlighted that dementia in LAC is a major public health issue with a predicted four-fold increase of its prevalence by 2050 (4, 5). This predicted growth, which is partially due to the increase in life expectancy (6), calls for better diagnostic procedures. The underdiagnosis of dementia in LAC remains a challenge (2). Barriers to diagnosis in the region include inadequate training (7, 8), especially among primary care physicians (2, 9), together with insufficient access to both healthcare and specialized services such as neuropsychological assessment (2, 6).

Epidemiological studies from the LAC region are scarce and existing evidence is limited, nevertheless making only modest contributions to global prevalence figures (10). Most of the literature available on the epidemiology of dementia comes from North American and European cohorts. The most extensive studies on the prevalence of dementia in LAC countries identified frequency rates similar to those reported by western and eastern countries (11–13). Among neurodegenerative dementia, Alzheimer's disease dementia (ADD) and Lewy body dementia are the leading cause of dementia, following by Frontotemporal dementia (FTD), the third most common form of dementia across all age groups, after, and is a leading cause of early-onset dementia (14, 15), with a prevalence ranging from 3 to 26% described in North America and European populations (16, 17).

FTD is an insidious neurodegenerative clinical syndrome characterized by progressive deficits in behavior, executive function, and language (16, 18, 19). FTD is often underdiagnosed, due primarily to the lack of awareness as well as clinical overlap with psychiatric disorders (15, 20). Although the impact of FTD on LAC countries seems to mirror that of developing countries, barriers to the diagnosis of and post-diagnostic support for this type of dementia differ across such countries (2, 3).

Regarding clinical diagnosis, as mentioned, FTD is often underdiagnosed (15, 20, 21). FTD symptoms typically start between the ages of 40 and 65 in the majority of cases, but it can also occur in younger and older individuals (16, 22). In LAC, the most common approach is to rely solely on clinical criteria for diagnosis. Unfortunately, for many clinical subtypes of FTD, there is only a modest correlation between the clinical features and the underlying neuropathology of the disease. Other diagnostic support such as specialized neuropsychological services and/or structural and functional neuroimaging studies are less readily available in the region (2). These well-known limitations have traditionally led to a higher rate of missed diagnosis and when is posed to significant delay in FTD diagnosis, which increases the subsequent burden on caregivers (23, 24). Pathologically, post mortem brains of people who had FTD are characterized with frontotemporal lobar degeneration (FTLD) and intracellular depositions of three main proteins: RNA-binding protein TDP-43 (~50%), microtubule-associated protein Tau (~40%), and, in rare cases, RNA-binding protein (FUS, 5%) (25). Importantly, FTD has a strong genetic component, with up to 40% of cases having a family history of dementia, psychiatric disease, or motor symptoms, and 20–30% of cases having an autosomal dominant pattern (26, 27). Mutations in three major genes have been described: *C9orf72* (chromosome 9 open reading frame 72), *MAPT* (microtubule-associated protein tau), and *GRN* (progranulin) discussed below in this review.

Biomarkers, defined as a characteristic that is objectively measured and evaluated as an indicator of normal biological processes, pathogenic processes (28), have emerged as promissory methods to provide and/or complement clinical diagnosis for this complex syndrome, although most evidence is needed to incorporate most of them in the routine clinical practice (29–31). Biomarkers have been currently classified in three main topics including genetic, neuroimaging, and fluid biomarkers (28, 32). In FTD, diagnostic biomarkers could help discriminate between individuals with FTD, control individuals, and individuals with other neurodegenerative diseases including ADD, as has been described in LAC cases (3). Biomarkers could also help differentiate between clinical, genetic, or pathological subtypes. Other biomarkers could also be used to tailor pharmacological treatment or determinate prognosis (30).

The study of biomarkers in FTD requires sophisticated procedures that only a few research centers have access to in LAC. Moreover, biomarker measurements or research are not funded by public health (2, 3). In this scenario, new peripheral biomarkers constitute a promising possibility to implement, for e.g., fluid biomarkers because of their accessibility, reduced cost, and easy management in our LAC region. Nevertheless, the use of biomarkers from fluids is also scarce, currently assessed only for research purposes (3). In addition, neuroimaging techniques are the most expensive and least available, only accessible in specialized medical centers in large cities in LAC. Furthermore, the reliability of available biomarkers, not only in LAC, is limited to centers specialized in dementia, such as memory clinics, where there is more experience in the accurate diagnosis of dementia. Another barrier present in LAC countries is that their validity has not been studied in native populations of each country, given the existing ancestry and genetic mix that represent each LAC country (1, 2). For example, genetic studies in Latino, mixed, or indigenous populations represent only 3% of studies of polygenic risk (3).

Considering the impact of FTD on LAC and the barriers to diagnosis of this progressive neurodegenerative disease, the advent of promising biomarkers (genetic, neuroimaging, and fluid-based) that can enhance diagnostic accuracy and help overcome outstanding needs could have a significant impact on this region. This review aims to update the knowledge base on biomarker development for FTD, with an emphasis on published studies from LAC and highlights the need for further development of FTD biomarkers that can be generalized to broader settings and diverse populations.

## Materials and Methods

### Database Search

A systematic search of the online literature was carried out targeting journals indexed by PubMed Central, Redalyc, Scopus, and SciElo databases. Pub-Med Central corresponds to the digital archive of the United States National Institutes of Health and it was selected for its scope and importance in the biomedical and life sciences; this database allows access to free material but the use of the material is subject to copyright and/or license terms. Redalyc is an academic project promoted by the Autonomous University of Mexico, in collaboration with other institutions, for the dissemination in Open Access of the scientific publishing activity that occurs in and on Ibero-America; and it was selected for its reach in regional populations. Scopus is a bibliographic database of abstracts and citations of scientific journal articles, which are peer-reviewed. This database was selected for its antiquity (1966) and scope since it is sponsored by Elsevier. SciELO is a Brazilian project that promotes the development and operation of Latin American collections for all areas of knowledge, with publications preferably in English, but also in other languages. It was elected for indexing many national and Latin American journals.

To identify potentially eligible studies related to FTD cognitive dysfunction biomarkers, the PRISMA Checklist and PRISMA Statement for Reporting Systematic Reviews and Meta-Analyses was followed, to have a validated and consensual research methodology (33). Two of the authors (MFA and PO) independently searched for articles associated with the following keywords in English: [(Biomarkers) AND (dementia)] OR [(Biomarkers) AND (frontotemporal dementia)] OR [(Biomarkers) AND (frontotemporal dementia behavioral variant)], and then, the procedure was reproduced with the same keywords, translated to Spanish and Portuguese. Those languages were selected because they correspond to the main languages used in Latinamerica, therefore ensuring to include all the Lan American research. Other languages such as french or german were not included, since those papers, despite having Latin American authors, were most probably not based on Latin American population. Initially, the search keys used considered the other clinical patterns of FTD, however in previously exploratory search, only the behavioral variant showed results based on the systematic review formula, for which it was decided to limit the search to the behavioral variant.

### Eligibility Criteria and Study Selection

Studies were considered eligible for data extraction if they meet the following inclusion criteria: original peer-reviewed articles (empirical, quantitative, longitudinal studies, follow-up studies, neuroimaging studies, randomized controlled trials, quasi-randomized controlled trials, cross-sectional studies, longitudinal studies) written in Spanish, English or Portuguese; published between January 2000 until November 2020, based on human Latin American populations, considering samples with FTD pathological characteristics, which their contents were about genetic, neuroimaging and fluid biomarkers. If some of the results found were still in press and could be checked by title and abstract, they were included too for the full-text review, by contacting the authors. No particular diagnostic criteria were required for the samples to be included because the main objectives of some potential results might be comparing them.

The exclusion criteria considered were: studies with no LA population samples, studies conducted with non-human animals, and studies written in a language other than those previously referred. We considered excluded from our investigation model: prospective studies, interview studies, retrospective studies, clinical and treatment trials, qualitative studies, mathematical modeling, experimental replications, scientific simulations, field studies, focus groups, non-clinical case studies, literature reviews, systematic reviews, and meta-analyses.

### Process of Selection

**Figure 1:**
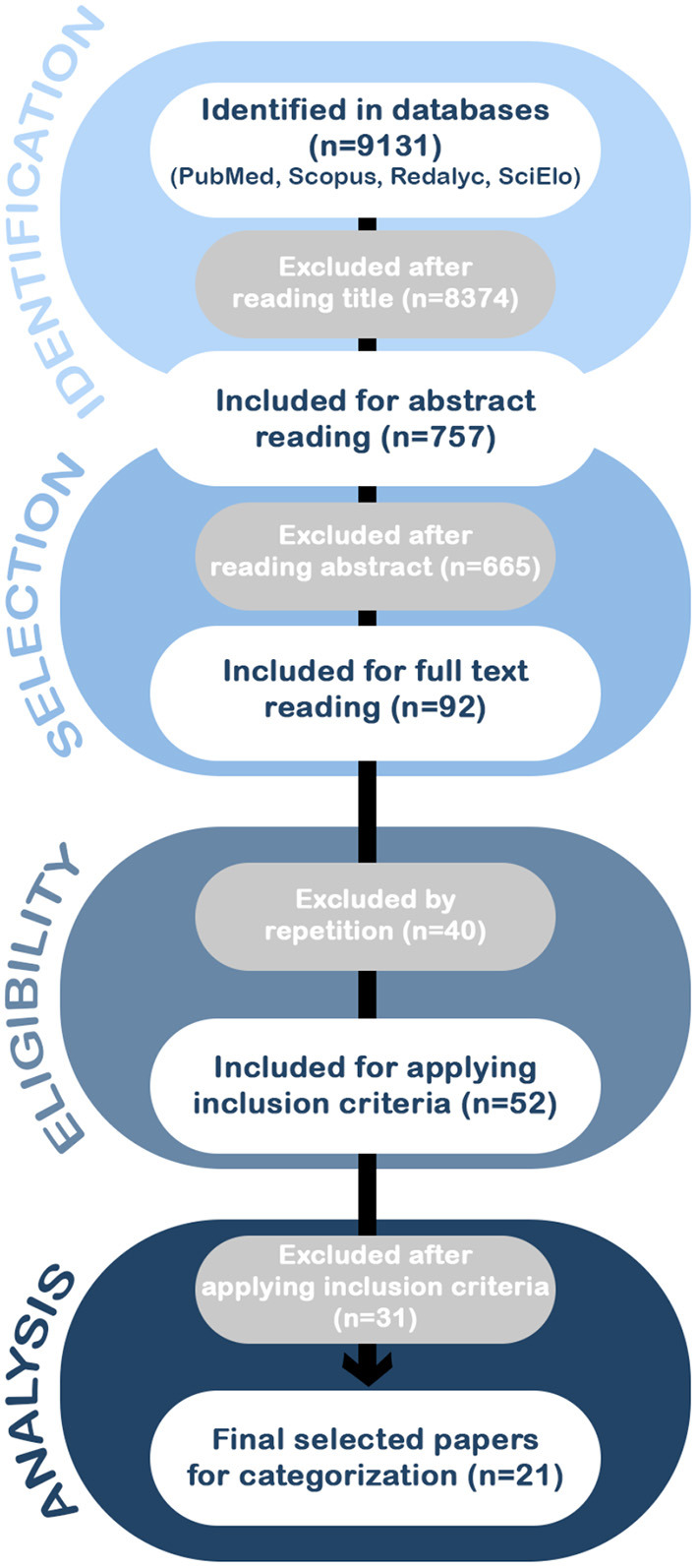
The flow of information through the different phases of the systematic review according to the PRISMA statement. The search of PubMed, Scopus, Redalyc, and SciElo databases provided a total of 9,131 citations. Of these, 8,374 studies were discarded after reviewing the titles, and of those, 665 abstracts did not clearly meet the criteria. After adjusting for duplicates 52 of the 92 articles remained. The full text of the remaining 52 citations was examined in more detail. It appeared that 31 studies did not meet the inclusion criteria as described. No unpublished relevant studies were obtained, achieving a selection of a total of 21 articles for the analysis.

In the first stage, MFA and PO searched for the selected keywords using boolean operators. With these first results, in a second stage, the articles were reviewed and selected according to their titles; from here, those who met the search words and/or the eligibility inclusion criteria were considered for the next stage. In the third stage, the abstracts and the full texts were read by PO and MFA to ensure papers met the criteria for sample descriptors, language, type of study, and contents. When the third stage was completed, the exhaustive review of each paper for the final selection was made by three authors (MA-P, PO, and TL). Disagreements were resolved by discussion between the three authors and, in case of disagreement, a fourth opinion was sought from the other authors for a final decision. Finally, the resulting sample of papers was divided between the other authors for the data analysis and synthesis. The flowchart in Figure 1 illustrates the sequence of actions and outcomes.

### Data Synthesis

MFA recorded specific data for each study (Table 1) including first all relevant citation information [author name(s), year of publication, Digital Object Identifier (DOI), and the data-based site where the article can be found] to facilitate the individual search for readers. Secondly, the country from which the study sample was recruited is reported with the purpose of highlighting research status in different localities. Third, each paper was reviewed and classified by the general biomarker technique used in its methods (category in the table): biomarkers (fluid-based), neuroimaging, and genetics. This was done to help readers categorize the amount of information available for each modality. The specific technique used in each category was presented in the specification column, highlighting the methodological approaches most often used in Latin American and Caribbean research. Finally, if the selected article provided information about the connection of said biomarkers to a particular cognitive domain, this was reported in the cognitive column. This data summary provides a high-level overview of the research occurring in LAC and allows reflection on the utility of biomarker information and translational research to the biomedical field.

**Table 1:** Papers resume table.

**Authors**	**Year**	**DOI**	**Country**	**Category**	**Specifications**	**Cognitive-domain associated**
Baez et al. (34)	2016	10.1016/j.cortex.2015.11.007	Colombia, Argentina, Chile	Neuroimaging	MRI-VBM	Social cognition
Baez et al. (35)	2016	10.1159/000441918	Colombia, Argentina, Chile	Neuroimaging	MRI-VBM	Social cognition
Bachli et al. (36)	2020	10.1016/j.neuroimage.2019.116456	Colombia, Argentina, Australia	Neuroimaging	Machine learning	Executive functions
Baldeiras et al. (37)	2015	10.1016/j.jns.2015.09.022	Brazil	Fluid Biomarkers	Aβ42/Tau ratio	Unspecified
Bertoux et al. (38)	2018	10.3233/JAD-170771	Francia, Chile	Neuroimaging	Visual atrophy ratings and VBM	Episodic memory
Cintra et al. (39)	2018	10.1016/j.neurobiolaging.2018.01.007	Brazil	Genetics	C9orf72	Syntomatic ALS, FTD and MND presentation
de Souza et al. (40)	2019	10.1590/1980-57642018dn13-030015	Brazil	Neuroimaging Fluid Biomarkers	PET-FDG, Aβ42, Tau, P-Tau in CSF	Executive functions
Dottori et al. (41)	2017	10.1038/s41598-017-04204-8	Argentina, Colombia	Neuroimaging	Resting-State: weighted symbolic dependence metric	Unspecified
Fernandez Suarez et al. (42)	2016	10.1080/13554794.2016.1186700	Argentina	Genetics	C9orf72	Unspecified
Fraga et al. (43)	2019	10.1016/j.neuroscience.2019.09.008	Brazil	Fluid biomarkers	hsCRP, IL-1β, IL-6, TNF, TGF-β1, AnxA1 and LXA4 in blood and CSF	Unspecified
Gatto et al. (44)	2017	10.1016/j.neurobiolaging.2017.02.002	Argentina	Genetics	MAPT	Executive functions attention
Itzcovich et al. (45)	2016	10.1016/j.neurobiolaging.2016.02.001	Argentina	Genetics	C9orf72	Unspecified
Miranda et al. (46)	2017	10.4067/s0034-98872017000700896	Chile	Genetics	C9orf72	Language and motor
Moguilner et al. (47)	2018	10.1038/s41598-018-29538-9	Argentina, Colombia	Neuroimaging	Resting-State: weighted symbolic dependence metric	Unspecified
Niikado et al. (48)	2019	10.1093/gerona/gly179	Argentina	Neuroimaging Fluid Biomarkers	MRI, cortical thickness, NfL in CSF	Unspecified
Riudavets et al. (49)	2013	10.1111/bpa.12051	Argentina	Genetics	PS-1	Unspecified
Santamaria-Garcia et al. (50)	2016	10.3233/JAD-160501	Colombia, Argentina, Chile	Neuroimaging	VBM	Neuropsychiatric symptoms
Santos et al. (51)	2014	10.1016/j.pnpbp.2013.06.019	Brazil	Fluid biomarkers	PBMC	Unspecified
Santos et al. (52)	2020	10.1016/j.jpba.2020.113424	Brazil	Fluid biomarkers	Plasma metabolite profile with GC-MS	Unspecified
Sedeño et al. (53)	2017	10.1002/hbm.23627	Colombia, Argentina, Australia	Neuroimaging	fMRI and graph-theory	Unspecified
Takada et al. (54)	2016	10.1097/WAD.0000000000000153	Brazil	Genetics	MAPT and GNR	Unspecified

*Articles used for the data analysis, showing an organization following an order by authors, year, digital object identifier (DOI), country, category, specification, and cognitive domain associated*.

## Results

After performing the PRISMA analysis, our search identified 21 studies on FTD and biomarkers in the LAC region. The selection process is depicted in the flowchart in Figure 1.

### Biomarkers

A biomarker is defined as an objectively measurable indicator of a biological state or pathological condition. A biomarker must be reproducible, stable, available to a large part of the population and reflect relevant disease processes (28). Biomarkers have the potential to be useful in dementia in several ways, including distinguishing different aspects of underlying pathology, detection of pre-symptomatic pathological changes, predicting decline or conversion between clinical disease states, and monitoring disease progression and response to treatment (32). As mentioned, the diagnosis of FTD is particularly challenging because the relationship between clinical symptoms, pathology, and genetic causes are complex (31, 55, 56). In this scenario, biomarkers represent a potentially informative diagnostic tool for this condition. However, almost all biomarker studies in FTD have been performed in North American and European populations (57), neglecting LAC countries (3). Here, we provide a brief update and the current state of knowledge on genetic, neuroimaging, and fluid biomarkers for FTD in the LAC region.

### Genetics Biomarkers for FTD in LAC

A strong genetic component has been observed in FTD, where 20–30% of cases have an autosomal dominant inheritance (26, 27, 58). This inheritance is mainly due to mutations in the genes *C9orf72, GRN*, and *MAPT* (59). Mutations in *MAPT* and *GRN* each account for 5–11% of total FTD cases (26). In 2011, a novel pathogenic expansion intronic to the gene *C9orf72* was identified, which has subsequently been found to be the most common genetic cause of FTD in Northern Europe and North America (60–62). In addition, mutations have been identified in other genes such as *VCP, CHMP2B, TARDBP, FUS, EXT2, SQSTM1, CHCHD10, TBK1, OPTN, CCNF, TIA1* in rare cases of FTD (63).

#### C9orf72

A hexanucleotide repeat (GGGGCC, G_4_C_2_) expansion in chromosome 9 open reading frame 72 (*C9orf72*) [GenBank: JN681271] was discovered to likely be the most frequent genetic cause of bvFTD, FTD with motor neuron disease (FTD-MND), and amyotrophic lateral sclerosis (ALS) in some populations (60, 62). In Europe and North America, the *C9orf72* expansion accounted for nearly 40% of familial and 8% of sporadic ALS, as well as 25% of familial and 6% of sporadic FTD cases (64). In contrast, the frequency was extremely rare in Asian (65, 66) and Middle Eastern countries (67).

Regarding the genetic situation in the LAC, some studies of *C9orf72* have been described in Chile, Argentina, and Brazil. In Chile, a case report of a family carrier of *C9orf72* mutation affected by non-fluent aphasia leading to mutism and mild parkinsonism was described (46). In Argentina, the first case with FTD and *C9orf72* mutation was reported in 2016 (42). A Brazilian kindred with FTD and FTD-ALS was reported in 2012, in which significant heterogeneity across different family members was seen and subtle behavioral changes were observed decades before a diagnosis of bvFTD was made (68). Later, the first characterization of *C9orf72* expansion in a group of patients was carried out in Latin America (45). Thirty-three patients with FTD and 50 patients with Amyotrophic Lateral Sclerosis. Hexanucleotide expansion was identified at a frequency of 18.2% in the FTD group while expansion explains 37.5% of the familial cases. In the group with ALS, the expansion was identified in 1 patient with a family history of the 3 cases studied, while in sporadic ALS the expansion was identified in 2.1% of the patients (45). In Brazil, a group of 404 patients with ALS and 67 with FTD were assessed for*C9orf72* pathogenic expansion. Pathogenic repeat expansions were found in 11.8% of familial ALS and 3.6% of sporadic ALS. In the cases of FTD, the pathogenic expansion was identified in 7.1% of the familial cases and was not detected in sporadic cases. Among the 35 cases of ALS with the *C9orf72* mutation, 25.7% also presented clinically with FTD; and among the 15 FTD mutation carriers, 20% also had ALS (39).

#### MAPT

Microtubule Associated Protein Tau (*MAPT*) encodes tau proteins involved in microtubule stabilization and assembly. Mutations in this gene cause tau splicing alterations, promote tau cytoplasmic aggregation, or cause tau hyperphosphorylation, which generates microtubule instability (18, 69). Mutations in *MAPT* in FTD have been reported at 17.9% in a British study and 4.7% in a French study (70, 71). Interesting, *MAPT* mutations were absent in Korean and Indian cohorts (72, 73). Regarding LAC status, in Argentina, a missense mutation p.P301L in exon 10 of the *MAPT* gene has been described in a large family with a behavioral variant of FTD (44). In Brazil, 55 patients with behavioral variant FTD, 11 with semantic variant PPA, and 10 with non-fluent variant PPA were studied. In that study, *MAPT* mutations were found in 7.1% of the entire cohort and in 10.5% of the familial cases (54).

#### GRN

Progranulin protein is encoded by the *GRN* gene and is expressed in a wide variety of cell types both in the periphery and in the central nervous system (74). This protein has several functions including activation of signaling cascades for neuronal growth, inflammation, and wound repair (18, 19, 74). The frequency of *GRN* mutations in FTD has been reported to be 3–15% in studies in North America and Europe cohorts (60, 70, 71, 75–78), while in Asia, the frequency was 0–1.6% (72, 73, 79). Among family cases, frequencies of 24.8% have been described in northern Italy, 20% in the UK, and 14% in France (70, 71, 75). In Brazil, the same cohort described above also assessed *GRN* and identified mutations in 9.6% of the total cases, including 31.5% of the familial cases, making *GRN* mutations the most common form of monogenic FTD in that sample (54).

#### TARDBP

Gene codified for a protein called transactive response DNA binding protein 43 kDA (TDP-43). This protein has functions such as RNA transcription, splicing, transport, and stability (80–82). Mutations in TARDBP are not common. Mutations in TARDBP are identified mostly in familial ALS patients, but also in sporadic FTD, AD, and PD cases (83–87). In Brazil, Machado-Costa identified a TARDBP mutation in a 54-year-old patient diagnosed with semantic dementia. This mutation was identified in the exon 6 of TARDBP corresponding to a p.I1383V mutation (88).

#### Presenilin-1

PSN-1 gene is frequently mutated in familial AD (89, 90), however, some mutations in this gene can be associated with an FTD phenotype (91). PSN-1 mutations may be associated with FTD phenotype in a minority of cases (91, 92). An Argentine family with FTD history was studied and was identified with the M146V mutation in PSN-1. This family showed histopathological changes of both Pick's disease and AD (49).

#### TREM2

Homozygous or compound heterozygous mutations of TREM2 have been associted to Nasu-Hakola disease which is characterized by bone involvement with an early-onset FTD phenotype (93, 94). These mutations of TREM2 have also been associated with FTD-like presentations without bone involvement (95, 96). Patients with FTD-like syndromes have been identified harboring homozygous or compound heterozygous mutations in TREM2 including p.Q33X, p.Y38C, p.R47C, p.R62C, p.T66M, p.D86V, p.D87N, p.D134G, among others (93, 95–100). Also, for heterozygous mutations in TREM2, association studies have been performed to determine the conferred risk of each variant. Two meta-analyses of rare variants in TREM2 found that the p.R47H and p.T96K variants confer a 2- to 3-fold increased risk of FTD in European populations (101, 102). In a Colombian family that presented the bvFTD phenotype (and no bone phenotype) was identified TREM2 p.W198X mutation in homozygosity. The clinical phenotype identified in the Colombian family with homozygous TREM2 mutations suggests that the genetic basis of monogenic bvFTD in LAC may be more heterogeneous than the families observed in northern European populations (103).

### Neuroimaging and Neurocognitive Studies in FTD

Classically, FTD cases show frontotemporal and insular atrophy in structural neuroimaging, with Magnetic Resonance Imaging (MRI) (104). In functional neuroimaging including positron emission tomography (PET) or single-photon emission computed tomography (SPECT), hypometabolism and hypoperfusion have been described (105), suggesting the involvement of either structural and/or functional impairment of the frontal lobe in the pathogenesis of FTD (19). Recent advances in the study of neuroimages have incorporated new modalities such as diffusion tensor imaging (DTI), resting-state functional MRI, arterial spin labeling (ASL), and tau PET imaging, allowing investigation of connectivity and molecular changes in different clinical populations (105). Aiming to understand the application of neuroimaging in FTD, several LAC teams have described novel techniques to further understand the underlying pathology of FTD and to help in the differential diagnosis. Here, we describe how neuroimaging has allowed us to study the neural bases of cognitive deficits in FTD using different techniques.

### MRI Studies

#### Structural

The neuroanatomical correlates of different cognitive tasks were used to evaluate specific symptoms of FTD, to look for the pathogenic substrate of that clinical manifestation. A multinational team of researchers, including participants from Chile and France, aimed to identify and discriminate the structural anatomical markers of episodic memory impairment in bvFTD, comparing those patients with AD patients and healthy controls, finding that impairment of medial/lateral temporal atrophy is associated with memory deficits (38).

Social cognition deficits seem to be a critical marker of the disease. Reports from LAC in this domain have shown neurocognitive deficits in FTD related tofacial emotion recognition (106–109), empathy (34, 110–112), theory of mind (106, 107, 112), moral judgment (35, 113), moral emotions (114), and interoception (115).

A multinational team of researchers from Argentina, Chile, and Colombia looked for a structural correlate of the moral judgment impairment often seen in bvFTD, finding that in bvFTD patients, judge harm permissible had an inverted relationship with the gray matter volume in the precuneus, thus implying that processing intentions and outcomes for moral judgments rely on regions beyond the Ventromedial Pre-frontal Cortex (35). The same group also described that in bvFTD patients, impairment in intentionality comprehension was associated with atrophy on limbic structures like the amygdala and anterior paracingulate cortex, while impairment in empathic concern was associated with atrophy of the orbitofrontal cortex. This is one of the first LAC studies to provide a structural base for the core neurocognitive deficit in FTD (34). The aim of the previous study was mainly to find a structural correlate of symptoms. No description of the accuracy of these methods was described, to use it, for example, as a diagnostic biomarker. However, the authors propose further research is needed and could eventually have other uses, such as diagnosis clarification (34).

The contextual fluctuation different social abilities seems to be a hallmark of FTD (116–120), reported impaired in FTD populations from LAC (121, 122). Many of these contributions from LAC have evidenced a multi-feature framework of social cognition in FTD, connecting behavior, electrophysiology, and multimodal neuroimaging (50, 53, 115, 123–126).

Research that used machine-learning algorithms (computational-decision methods) to identify bvFTD and AD, was carried out by a team from Argentina and Colombia, in collaboration with one team from Australia (36). This team was the first one to validate the importance of cognitive-behavioral assessment and neuroanatomical measures combined to identify bvFTD and AD from controls (36). In addition, the combined methods showed high rates of classification (>91%) and prediction (>91%) of AD and bvFTD in new cohorts. These results demonstrate the importance of the application of computer methods combined with cognitive screening assessment (global cognition and executive function) and brain atrophy volume (voxel-based morphometry from fronto-temporo-insular regions in bvFTD) (36).

#### Functional Connectivity

In the field of neuroimaging, functional connectivity is a very sensitive tool that is becoming increasingly popular. Functional connectivity is defined by Friston “as the temporal coincidence of spatially distant neurophysiological events” (127). In LAC, this technique had no gold standard reported until a group from Argentina conducted a multicenter analysis of functional imaging in bvFTD (53). Their multidimensional approach involved fMRI and Graph theory to yield a gold-standard that can aid in the distinction between bvFTD and healthy controls. To evaluate Functional connectivity several analyses were performed: seed analysis, inter-regional connectivity, and graph-theory approaches. They found interesting results indicating that frontal and temporal areas showed less integrated and interconnected areas in FTD as described by Freeman “indicate the number of shortest paths that pass through a node and link the other node pairs across the network” (128). In addition, the authors showed in 148 patients that graph-theory based on weighted matrices could distinguish between bvFTD and other neurodegenerative diseases across centers, highlighting this technique as a potential gold standard to analyze brain networks in bvFTD. Moreover, betweenness centrality and graph theory are both methods able to detect brain connectivity abnormalities and discriminate bvFTD from healthy controls (53).

### Summary of Neuroimaging and Neurocognitive Studies

The research for new and early biomarkers for neurodegenerative diseases, such as FTD, is one of the main goals of many research groups. All the presented research related to neuroimaging has very high relevance. Search for biomarkers for early diagnosis of neurodegenerative disease is pivotal and neuroimaging methods are potential sensitive biomarkers for being used in the population of the LAC region. The majority of the LAC research described in our review is based on structural neuroimaging, and functional imaging. Those biomarkers appear to be more affordable in the LAC context and further research is needed to expand these biomarkers across LAC, allowing a better diagnosis in a limited budget context. Nevertheless, several other biomarkers are being used around the world, including functional imaging allowing *in vivo* imaging of proteins, DTI allowing to evaluate the connection between lobes, among others (105). They have provided important insights into FTD pathology, especially in HIC (104, 129), therefore an effort should also be done to increase the access to those resources for special cases.

### Electroencephalographic Studies

A multicenter study, conducted by a team from Argentina and Colombia, developed a novel non-linear association method to evaluate the ability to identify patients with bvFTD and healthy controls based on resting-state functional connectivity. This method called weighted Symbolic Dependence Metric (wSDM) inspired by EEG studies and based on machine learning, proved to be superior to linear measurements (R Pearson) widely used in the identification of functional connectivity in patients with bvFTD (41, 47). Another similar non-linear connectivity method has been proven robust to classify FTD patients based on the dynamical fluctuation assessed with machine learning (130). This study also provided evidence of generalization of classification to both LAC and High-Income Countries (HIC) datasets. Although few studies with EEG were founded, EEG is a cheap and accessible method of research, especially useful for LMIC like in Latin America.

### FTD-Related Fluid Biomarkers

Cerebrospinal fluid (CSF) and blood are the most frequent fluids which have been described or studied as a diagnostic tool in dementias (131). Here, we will provide the most recent knowledge of the use of fluids biomarkers in LAC cohorts suffering from FTD.

#### Neurofilament Light Chain

NfL is a component of the neuronal cytoskeleton, which is involved in structural support, transport, and neurotransmission in neurons (132). NfL is released into the CSF and blood when neurodegeneration occurs (132). Increased levels of NfL have been reported in the CSF of patients with ALS and FTD (133, 134). NfL has been suggested as a marker of FTD severity, as high concentrations in CSF are associated with shorter survival (135). A strong correlation has been observed between plasma NfL concentrations and CSF (136, 137), and it has been shown that serum or plasma NfL levels are increased in FTD, reflecting disease severity and predicting clinical deterioration and brain volume loss (138–141). NfL concentration only increases during the symptomatic phase, while pre-symptomatic levels are usually similar to controls (142). NfL is also a promising blood biomarker in genetic frontotemporal dementia (*GRN, C9orf72*, and *MAPT*) (143). In a longitudinal study across people from Canada and Europe with pre-symptomatic and symptomatic genetic frontotemporal dementia, NfL levels showed changes over time and correlated them with longitudinal imaging and clinical parameters. During the study, NfL levels were increased in persons who converted from pre-symptomatic, highlighting serum NfL as an easily accessible biomarker in genetic FTD dementia (143). Another study using a meta-analysis approach of fluid biomarkers to differentiate DFT from AD described that NfL were useful in distinguishing both diseases (144–146). The only report about FTD and NfL in the LAC region was done in Argentina, where 13 patients with bvFTD, 6 with lvPPA, 2 with svPPA, and 4 subjects with nfvPPA were studied. NfL levels in CSF in patients with bvFTD are higher than in MCI, AD, and controls (48), which has been described in other studies (145, 147, 148).

#### Progranulin

Progranulin is a pleiotropic growth factor that is expressed in multiple tissues and cell types throughout the human body, serving important roles in normal tissue development, proliferation, regeneration, inflammation, and tumorigenesis (149, 150). In the brain, progranulin is involved in both neuronal survival and neurodegenerative disease (74, 151). Mutations in *GRN* cause disease through haploinsufficiency and CSF and plasma progranulin concentrations are reduced in *GRN* mutation carriers (152). Central nervous system progranulin levels are regulated differently from peripheral progranulin levels in neurodegenerative diseases (134, 153–157). This has also been observed in healthy elderly subjects (155). Peripheral levels may not adequately represent progranulin levels in the central nervous system (155, 156). Very low plasma progranulin levels have been observed in FTD patients with *GRN* mutations compared with sporadic FTD (152, 158, 159), suggesting that this analysis is useful for detecting carriers of *GRN* mutations that cause haploinsufficiency (160). The only study using *GRN* in LAC was done in Brazil (54). Plasma progranulin were evaluated in 7 GRN mutation carriers, 55 non-carriers mutation and 60 healthy controls. Levels of plasma progranulin were significantly lower in the FTD group carriers of *GRN* mutations than in the FTD group without *GRN* mutations or in the control group. Plasma progranulin levels were also lower in the FTD without GRN mutations group, in comparison to the control group (54).

#### TDP-43

TDP-43 is a protein involved in alternative splicing and transcriptional regulation (161). In ALS and FTD, TDP-43 protein suffers ubiquitination, hyperphosphorylation, and also truncation of C-Terminal, increasing its aggregation profile leading to neurotoxicity and further cell death (19, 25). Elevated levels of TDP-43 have been observed in CSF in patients with ALS and FTD, with higher concentrations in ALS than in FTD, suggesting that TDP-43 is a biomarker in this disease (162). This could be explained by the higher percentage of TDP-43-related pathology in ALS (~97%), while in FTD a significant percentage is due to other (mainly tau deposits) pathologies (~45%) (162). Majumder et al. conducted the first meta-analysis showing that TDP-43 in CSF is significantly increased in patients with FTD-ALS and ALS (163). However, this difference is not observed in patients with FTD alone. These data suggest the use of CSF TDP-43 as a biomarker for ALS (163). Plasma TDP-43 has been useful in differentiating FTD patients with TDP-43-based pathology from those with tau-based pathology (164). However, no differences in TDP-43 concentrations have been identified between patients with FTD and AD (165). By analyzing the phosphorylated form of TDP-43 (pTDP-43) which is added in the brain, they have shown a good correlation between plasma protein levels and pTDP-43 depositions in the brain (165). High concentrations of pTDP-43 in plasma were observed in C9orf72 and GRN mutation carriers, while total pTDP-43 levels were observed to be decreased (166).

All together suggest that TDP-43 may be used as a biomarker in FTD. However, at present, using our PRISMA methods we did not find studies in LAC countries and research of TDP-43 as a biomarker is still missing.

#### Aβ-Amyloid, Tau, and P-Tau

In a recent study, CSF amyloid-beta (Aβ)1–42, total tau (T-Tau), and phosphorylated tau (p-Tau) ratios, showed their clinical utility for differentiating AD from non-AD neurodegenerative dementias, distinguishing AD from both bvFTD and semantic dementia (SD, sensitivities, and specificities of 80–90%) (167). In a similar study, low levels of the secreted form of Ab precursor protein (sAPPb) in CSF have been observed in patients with FTD compared to patients with AD and controls (168). Interestingly, the Aβ_42_/pTau181 ratio showed better differentiation between AD and FTD patients (169). This study was supported by two other investigations reporting increased sensitivity (80–86%) and specificity (82%) of the Aβ_42_/pTau181 ratio, suggesting that those proteins are the best biomarker subset to differentiate FTLD from AD (37, 170). The plasma levels of p-Tau181 were significantly higher in patients on the AD spectrum groups and FTD patients, with the highest level in the FTD group (171). In a recent study, plasma p-tau181 distinguished AD of DFT with an AUC of 100% (172). Another phosphorylated form of tau, p-Tau217, has been studied in AD and other neurodegenerative diseases such as bvFTD or PPA, finding an AUC of 0.92 with a specificity of 81% and sensitivity of 93% to differentiate between these variants of FTD and AD (173). In Brazil, CSF AD biomarkers were used to distinguish a case of a frontal variant of AD and behavioral variant frontotemporal dementia (40). Importantly, the patient fulfilled criteria for probable bvFTD, however, CSF biomarkers signature showing low Aβ42, high Tau, and high p-Tau established a diagnosis of the frontal variant of AD (40).

#### GFAP

Glial fibrillary acidic protein (GFAP) is a protein widely expressed by numerous cell types of CNS, including astrocytes (174, 175). GFAP, an established marker of astrogliosis in neurodegeneration (174, 175), have been recently described as a possible biomarker for FTD (176–179). Increased levels of GFAP have been reported in AD and ALS patients in both CSF and serum (57, 177). Previous studies of GFAP in FTD showed increased CSF levels in symptomatic patients, however, changes in this protein's levels in the blood have not been identified (177–179). In a recent study, GFAP concentration was analyzed in FTD patients carrying mutations in *C9orf72, GRN* and, *MAPT* in both symptomatic and pre-symptomatic subjects (176). Increased plasma levels of GFAP were only observed in *GRN* mutation carriers. In pre-symptomatic stages of the disease, elevated GFAP concentrations were correlated with lower cognitive test scores and lower brain volumes, suggesting that GFAP increases in late pre-symptomatic stages. In symptomatic stages, higher GFAP concentrations were associated with faster rates of atrophy, suggesting that GFAP could be associated with disease intensity, progression, and survival (176). In our LAC regions, no studies in GFAP levels have been performed.

#### Inflammatory Biomarkers

It has been suggested that immune activation may be an early cause of neurodegeneration (180) or that the addition or accumulation of tau or TDP-43 induces an increased cytotoxic response leading to chronic neuroinflammation (181–183). In FTD, the immune response is likely to be triggered by the accumulation of poorly folded tau proteins or TDP-43, or the deregulation caused by signals released by damaged neurons or the deregulation of mechanisms to remove poorly folded or damaged neuronal proteins. These processes lead to neurodegeneration (180, 184–186). Changes in inflammatory markers in blood, serum, and CSF have been reported in different FTD subtypes, suggesting that inflammatory factors play an important role in the pathogenesis of the disease (187). Biomarkers include some of the pro- and anti-inflammatory cytokines, chemokines, and secondary messengers that coordinate the immune response through regulation of innate and adaptive responses in the periphery (188, 189).

Patients with genetic and sporadic FTD share similar patterns of inflammation at CSF (179). Patients with FTD show overexpression of tumor necrosis factor (TNF) and transforming growth factor (TGF-b1) in CSF, as well as microglia activation in atrophic areas of the brain (190, 191). One study of sporadic DFT reported elevated CSF levels of TNF-α (192), while another study reported decreased CSF levels of IL-12 (193). Smaller studies reported elevated CXCL8 and IL-15 levels in the CSF (194, 195). However, all of these findings were not reproduced in a subsequent study (196). Reports of elevated CSF levels from TGF-β and IL-11 (192, 197) have not yet been reproduced or denied, while two studies have identified elevated CSF levels from CCL2 in sporadic FTD (194, 196). Progranulin appears to be involved in neuroinflammation and microglia activation (198–200). In a small cohort of *GRN* mutation carriers, an apparent CSF profile of elevated CXCL10 and decreased levels of TNF-α, IL-15, and CCL5 have been described (196). Another recently identified marker of neuroinflammation is soluble TREM2. TREM2 encodes a receptor expressed on immune cells that regulate phagocytosis. In the brain, TREM2 is expressed exclusively by microglia (201), and it has been suggested that TREM2 levels are a marker of microglia and neuroinflammation activity (202). In carriers of TREM2 mutations, sTREM2 CSF levels are decreased, suggesting a loss of function as a pathological mechanism (203). One study also found that CSF sTREM2 levels decreased in a larger cohort of patients with FTD, including carriers with *C9orf72* and *GRN* mutations (204).

Studies of circulating inflammatory biomarkers in patients with FTD are scarce even though blood samples are easier to obtain than CSF and also, the results have been inconsistent. One study shows that IL-6 levels are increased in FTD patients carrying *GRN* mutations when compared to pre-symptomatic carriers, suggesting an inflammatory response when FTD symptoms appear (205). In a cross-sectional study of patients with a mutation in the gene *CHMP2B* associated frontotemporal dementia, levels of inflammatory markers such as CCL4 IL-15, CXCL10, CCL22, and TNF-α were found increased and significantly associated with cognitive decline, suggesting a peripheral inflammatory response to neurodegeneration (206). In Brazil, Fraga et al. for the first time evaluated different proteins involved in the immune response in patients with FTD (43). The proteins evaluated in plasma were high sensitivity C reactive protein (hsCRP), TNF, IL-β1, IL-6, TGF-β1, LXA4, and AnxA1, and investigated changes in LXA4 e AnxA1 levels in CSF bvFTD patients. For AnxA1 alone, a reduction in plasma levels was demonstrated in bvFTD patients compared to AD and controls. However, no difference was observed between AD and bvFTD in CSF (43). Another study performed in Brazil analyzed a B7-CD28/CTLA-4 pathway that is an important immunological signaling pathway involved in the modulation of T cell activation. Forty-six patients were included in this study divided into three groups: 27 AD, 10 FTD, and 9 control patients. The FTD group was composed of 7 patients with bvFTD, 2 patients with progressive non-fluent aphasia, and 1 patient with semantic dementia. CTLA-4 expression showed a reduction in FTD patients compared to AD or control groups (R. R. 205).

#### Proteomics and Metabolomics

Unbiased mass spectrometry (MS) was performed and identified 20 differentially abundant proteins between symptomatic GRN mutation carriers and 24 non-carriers and 9 between 19 symptomatic and 9 pre-symptomatic mutation carriers. These results were validated in subjects symptomatic and pre-symptomatic mutation carriers of C9orf72 and MAPT, in addition to GRN carriers (143). A validation study performed by targeted mass spectrometry showed significantly lower levels of NPTXR, CHGA, VSTM2B, PTPRN2, and VGF in symptomatic GRN mutation carriers compared to pre-symptomatic and non-carriers. Four of the 5 protein decreases (NPTXR, VSTM2B, CHGA, and PTPRN2) were observed in symptomatic GRN carriers as well as symptomatic C9orf72 carriers, suggesting that these changes are not specific for GRN associated FTD. In MAPT mutation carriers, significant differences in protein concentrations were only found for NPTXR and CHGA. This suggests that may there be differences in pathophysiology in MAPT mutation carriers or it may be due to the smaller sample size (143). The results show that synaptic, secretory vesicle, and inflammatory proteins are dysregulated in the symptomatic stage in mutation carriers and may provide new insights into the pathophysiology of genetic FTD (143). One study performed in Brazil using gas chromatography coupled to mass spectrometry (GC-MS) included nine patients with bvFTD, 17 with AD and 15 cognitively healthy controls in the training set, whose data were validated on a testing set of 8 bvFTD, 14 AD, and 10 controls (52). Differences were identified when compared to the bvFTD and control groups, but not between bvFTD and AD groups. The bvFTD group showed decreased levels of plasma of metabolites related to glycine/serine/threonine, alanine/aspartate/glutamate pathways, and aminoacyl-tRNA biosynthesis when compared to controls. These results suggest that impairment of amino acid metabolism and the translation process may be present in bvFTD patients (52).

## Conclusions and Future Directions

**Figure 2:**
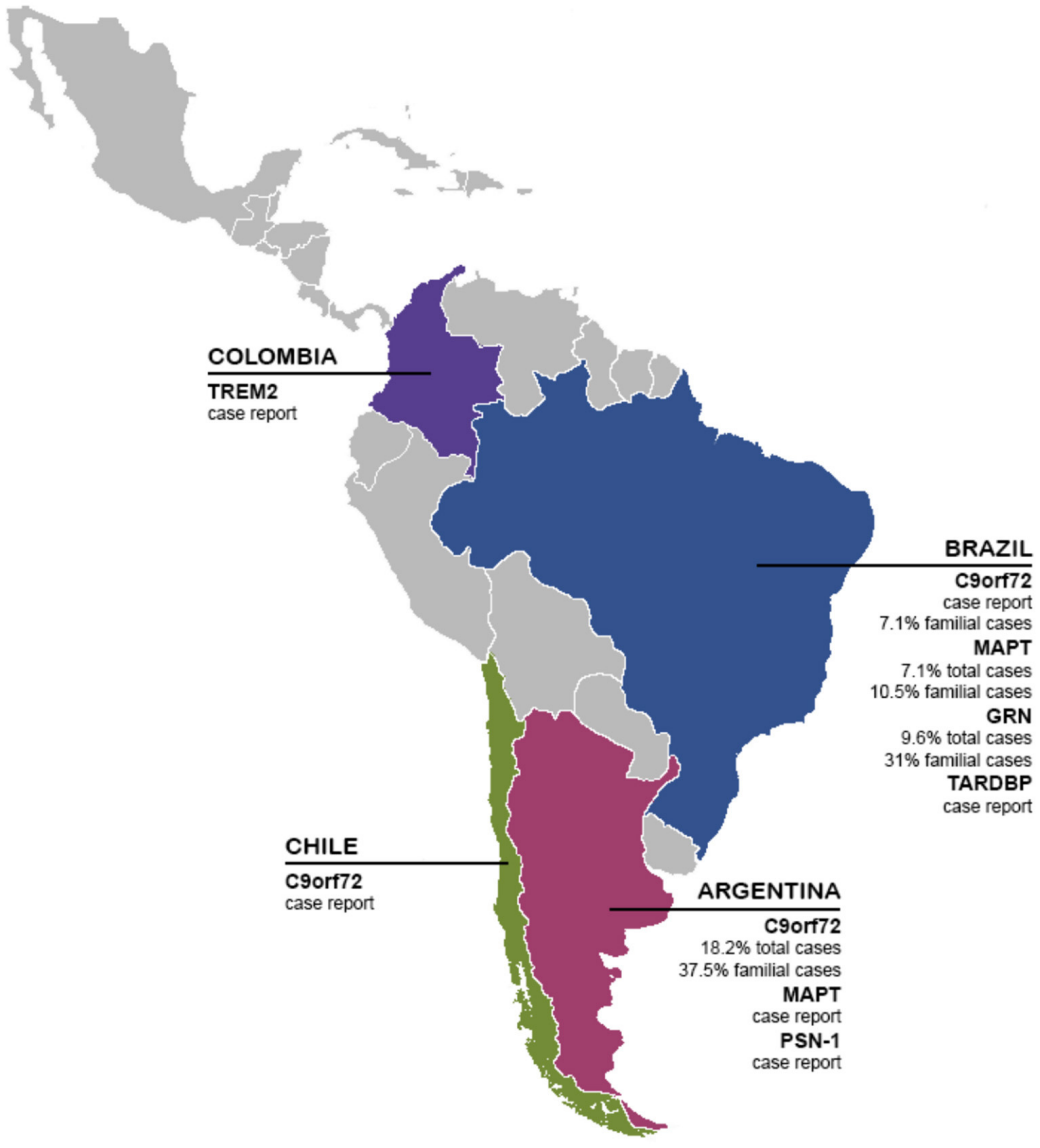
Genetics biomarkers of FTD in LAC. The presence and frequency of FTD genetic biomarkers (TREM2, C9orf72, MAPT, GRN, TARDBP, and PSN-1) in LAC.

FTD, like the rest of dementias, is a public health problem, often underdiagnosed, undertreated, and not fully understood. This situation is especially relevant in LAC, presenting several barriers to diagnosis, treatment, and further research on FTD. In this review, we showed local efforts to make research on biomarkers in the LAC region. Until today most of the knowledge about FTD comes from North America and Europe cohorts, providing guidelines and descriptions that do not necessarily capture the local reality in terms of psychopathology, genetics, or diagnostic tools. Aligned with that, our current analysis in this systematic review revealed only 21 articles published between January 2000 until November 2020 in LAC, considering FTD participants and genetic, neuroimaging, or fluid biomarkers studies (Table 1). Interestingly, most of the researchers are coming from Argentina, and Brazil, representing more than 55% of all of the manuscripts (Table 2). Most of the literature comes from genetics and neuroimaging studies, representing ~70% of the articles. As we showed in Figure 2, the *C9orf72* gene is widely represented in familial and sporadic cases from Chile, Brazil and Argentina, followed by *MAPT* and *GRN* genes, as described in HIC. Several neuroimaging techniques are being used, however, most of the LAC research described in our review is based on structural neuroimaging, functional imaging, and EEG. In this context, further research is needed to expand these biomarkers across LAC, allowing a better and accurate diagnosis.

**Table 2:** Quantity distribution of papers.

**Papers**	**Country**						**Category**		
	**Argentina**	**Brazil**	**Colombia**	**Chile**	**Australia**	**France**	**Genetics**	**Neuroimaging**	**Fluid biomarkers**
Quantity/Percentage (%)	12/35.3	7/20.6	7/20.6	5/14.7	2/5.9	1/2.9	7/30.4	10/43.5	6/26.1

*Quantity and percentage of papers by country and category for the data analysis*.

Important to emphasize, studies on fluid biomarkers also proceeded exclusively from Brazil and Argentina (37, 40, 43, 48, 51, 52). Nfl, PGNR, and TDP-43 proteins appear to be the best molecules for FTD diagnosis in most of the studies. However, no studies of TDP-43 in the LAC region have been performed to distinguish controls from dementia patients, making it clear that it is imperative to develop and study fluid biomarkers in our regions. Despite the contribution of LAC studies, our review suggests that biomarkers research is still needed to increase the comprehension knowledge about FTD pathology in LAC and their contribution to clinical diagnosis. Biomarkers research is yet limited in number with a small sample size or simply case reports. In a recent study, plasma p-tau181 distinguished AD of DFT with an AUC of 100% (172), suggesting that this protein could be used as a potential diagnosis tool, however no studies of this protein has been developed in the LAC region.

Knowledge of the clinical manifestation of FTD has progressed exponentially over the past 20 years (19). However, the heterogeneity of the clinical outcome of FTD together with the potential overlapping with other conditions leads to considerable misdiagnosis by clinicians (19). In context, clinicians, biomedical, and basic researchers have increased awareness about this disabling neurodegenerative condition, especially in vulnerable regions such as LAC. Moreover, considering the mixed genetic heritage of LAC and the high prevalence of cardiovascular risk (207), among others, we highlight the need to develop strategies to increase research in the region to study the contribution of biomarkers, mainly fluid biomarkers, to understand the pathology of FTD and improve diagnosis.

Recent years have seen a rapid development of biomarkers for FTD and other dementias (29–31). LAC region is experiencing increased demand for harmonized, innovative, and cross-regional studies on dementias, including FTD. Across the LAC countries, the case of FTD is even more challenging than AD. LAC region may be driven by unique genetic factors which could influence the prevalence and presentation of dementia (1–3, 208–213). However, region-specific determinants remain unknown and the region is still underrepresented in international publications/journals including studies in prevalence, social determinants, and local research of genetics and biomarkers (2, 3). Thus, specific knowledge on the regional reality of LAC is still scarce and limited (2, 3, 214). It is important to mention that the most frequent limitations raised by researchers are the lack of infrastructure, technology, availability of samples from native populations specific to each LAC country, and the high costs associated with biomarker analysis (3).

Recently, multiple regional research efforts have been developed in LAC countries focused on the use of machine learning for the combination of neuroimaging modalities as well as behavioral/cognitive assessment to a better understanding of different dementias in our region (36, 130, 215–219). A multi-feature framework, targeting no one single potential biomarker, but a multilevel combination of measures, tuned by machine learning algorithms robust to assess simultaneously multiple features, supporting redundancy of information, and extracting the main components via progressive feature elimination process, would represent a new-generation promissory approach to target the complex multimodal nature of FTD. Dementia research in the region is certainly reduced in comparison with HIC in the LAC, highlighting an urgent need to integrate different areas of dementia knowledge with a more global perspective (6, 209). Thus, the development of a more extended regional network establishing multi-center LAC initiatives is critical for global discovery and research standardization of dementia in these underrepresented cohorts.

## Data Availability Statement

The raw data supporting the conclusions of this article will be made available by the authors, without undue reservation.

## Author Contributions

CD-A and AS designed the proposal. PO and MA-P performed PRISMA analysis. CD-A, PO, TL, FH, VC, TE, MP, and AS wrote the drafts, discussed contributions from all co-authors. All authors searched the literature, participated in discussing the contents of the paper, contributed to editing, and approved the final version of the article.

## Conflict of Interest

The authors declare that the research was conducted in the absence of any commercial or financial relationships that could be construed as a potential conflict of interest.

## Abbreviations

Aβ: amyloid-beta peptides
AD: Alzheimer's disease
AnxA1: Annexin A1
bvFTD: behavioral variant of frontotemporal dementia
C9orf72: chromosome 9 open reading frame 72
COEP-UFMG: Ethics Committee of the Federal University of Minas Gerais
CSF: cerebrospinal fluid
EDTA: ethylenediaminetetracetic acid
FAST: Functional Assessment Staging
GRN: progranulin
hsCRP: high sensitivity C reactive protein
HWE: Hardy-Weinberg equilibrium
IATI: INNOTEST amyloid tau index
IL: interleukin
LX4: lipoxin A4
MAPT: microtubule-associated protein tau
MCI: mild cognitive impairment
NF: nuclear factor
NFTs: neurofibrillary tangles
PBMCs: peripheral blood mononuclear cells
PCR-RFLP: polymerase chain reaction-restriction fragment length polymorphism
SNP: single nucleotide polymorphism
SPM: specialized pro-resolving mediator
TDP-43: TAR DNA-binding protein 43
TGF: transforming growth factor
TNF: tumor necrosis factor.
